# Potential of miR-181a-5p and miR-630 as clinical biomarkers in NSCLC

**DOI:** 10.1186/s12885-023-11365-5

**Published:** 2023-09-12

**Authors:** Julija Simiene, Daiva Dabkeviciene, Diana Stanciute, Rimvile Prokarenkaite, Valerija Jablonskiene, Renatas Askinis, Kamile Normantaite, Saulius Cicenas, Kestutis Suziedelis

**Affiliations:** 1 National Cancer Institute, Vilnius, 08406 Lithuania; 2 Vilnius University Life Sciences Center, Vilnius, 10223 Lithuania; 3 Faculty of Medicine, Institute of Biomedical Sciences, Vilnius University, Vilnius, 01513 Lithuania

**Keywords:** miR-181a-5p, miR-630, Non-invasive clinical biomarkers, Response to treatment, NSCLC

## Abstract

**Background:**

The development of drug resistance and high mortality rates are the major problems observed in non-small cell lung cancer (NSCLC). Biomarkers indicating and predicting disease development towards these unfavorable directions are therefore on high demand. Many studies have demonstrated that changes in miRNAs expression may be associated with a response to treatment and disease prognosis, thus suggesting its potential biomarker value for a broad spectrum of clinical applications. The aim of the present study was to investigate the expression level of miR-181a-5p, miR-630, and its targets in NSCLC tumor tissue and plasma samples; and to analyze its association with NSCLC patient’s response to treatment and disease prognosis.

**Methods:**

The study was performed in 89 paired tissue specimens and plasma samples obtained from NSCLC patients who underwent surgical treatment at the Department of Thoracic Surgery and Oncology of the National Cancer Institute. Analysis of miR-181a-5p and miR-630 expression was performed by qRT-PCR using TaqMan miRNA specific primers. Whereas *BCL2*, *LMO3*, *PTEN*, *SNAI2*, *WIF1* expression levels were identified with KAPA SYBR FAST qPCR Kit. Each sample was examined in triplicate and calculated following the 2-^ΔΔC^t method. When the p-value was less than 0.05, the differences were considered statistically significant.

**Results:**

It was found that miR-181a-5p and miR-630 expression levels in NSCLC tissue and plasma samples were significantly decreased compared with control samples. Moreover, patients with low miR-181a-5p expression in tumor tissue and plasma had longer PFS rates than those with high miRNA expression. Decreased miR-630 expression in tumor was statistically significantly associated with better NSCLC patients’ OS. In addition, the expression of miR-181a-5p, as well as miR-630 in tumor tissue, are the statistically significant variables for NSCLC patients’ OS. Moreover, in NSCLC patient plasma samples circulating miR-181a-5p can be evaluated as significant independent prognostic factors for OS and PFS.

**Conclusions:**

Our findings indicate the miR-181a-5p and miR-630 expression levels have the potential to prognose and predict and therefore improve the treatment individualization and the outcome of NSCLC patients. Circulating miR-181a-5p has the potential clinical value as a non-invasive biomarker for NSCLC.

**Supplementary Information:**

The online version contains supplementary material available at 10.1186/s12885-023-11365-5.

## Background

Lung cancer is the most common neoplasm and a leading cause of cancer-related deaths. With an estimated 1,369,000 new cases per year worldwide, lung cancer is the second most common cancer type in men and women [1, 2]. Non-small cell lung cancer (NSCLC) is the most frequently diagnosed type of lung cancer, accounting for 80–85% of all lung cancer cases. Advanced forms of lung cancer are usually unresponsive to chemotherapy and five years survival rate is specific for only 10–15% of patients. Cisplatin and other cytotoxic agents are used as the first-line therapy; however, the development of drug resistance is a significant problem observed in NSCLC [3, 4]. The major clinical issue today is the absence of specific molecular markers for the disease prognosis, treatment prediction, and individualization.

Circulating miRNAs, so-called extracellular miRNAs, have gained attention as novel liquid biopsy targets. The identification of circulating miRNA is an attractive alternative to tissue biopsy for the diagnosis or characterization of cancer or other diseases [5]. miRNAs are short (21–23 nucleotides) non-coding RNA molecules that regulate the expression of proto-oncogenes and tumor-suppressor genes in a post-transcriptional level [6]. About 2650 mature miRNAs are recognized in human cells [7]. In mammals, miRNAs regulate approximately 30% of all protein-coding genes and are involved in main cellular processes such as growth, differentiation, proliferation, and apoptosis, related to immune response and tumor formation processes [8].

Due to new molecular biology techniques such as microarray or RNA sequencing, aberrant expression of various miRNAs was identified in NSCLC patients’ blood plasma and tissue samples [9, 10]. Study results suggest that miR-181a-5p and miR-630 may be involved in the development of cisplatin resistance and may also play a role in regulating drug resistance-related pathways. It has been shown that a high expression of miR-181a-5p increased the sensitivity of human lung carcinoma A549 cells to platinum-based chemotherapy drugs such as cisplatin, carboplatin, and oxaliplatin; whereas a high expression of miR-630 increases resistance to cisplatin, resulting in a decreased cell proliferation and the suppression of the signaling cascades that occur due to DNA damage [11]. Moreover, miR-181a-5p is downregulated in tumor tissue of all main lung cancer histological subtypes, suggesting that miR-181a-5p silencing is significant for tumor initiation and progression processes [12].

The gene encoding miR-181a-5p is localized on the long arm of chromosome 1, in a 31.3 region. miR-181a-5p belongs to the miR-181 family, which also includes miR-181b, miR-181c and miR-181d. Their sequence and secondary structure are relatively conservative. According to miRDB: the microRNA database, miR-181a-5p has 887 target genes, including *BCL2*, *LMO3*, *PTEN*, *SNAI2*, *WIF1*, which are related to tumor development, cell cycle control, signal transduction processes, and apoptosis [13, 14]. Altered expression of miR-181a-5p is observed in thyroid [15], breast [16], gastric [17], and colorectal cancer [18].

The gene encoding miR-630 is localized on the long arm of chromosome 15, in a 24.1 region. miR-630 has 182 target genes; among them are *PRKCI*, *CCNE2*, and *PIK3C2A*, which are involved in cell cycle control, proliferation, signal transduction, and oncogenic transformation. Moreover, as specified by miRDB: the microRNA database, *BCL2, LMO3, PTEN, SNAI2, WIF1* are also molecular targets of miR-630 and may be significant in NSCLC progression [19]. Expression changes of miR-630 are determined in patients with breast [20], gastric [21], ovarian [22], renal cell carcinoma [23], and colorectal cancer [24].

Studies have shown that changes in the expression of miRNAs and their target genes are associated with response to treatment and may be used as biomarkers in NSCLC treatment [25]. Bioinformatics analysis of Pubmed, Gene Expression Omnibus, and ArreyExpress databases showed that miR-181a-5p has the potential to be used as a biomarker in lung cancer [26]. Raw sequencing data and clinical information from The Cancer Genome Atlas showed that miR-181a is downregulated in lung squamous cell carcinoma [27]. Additionally, miRNA expression results based on miRNA next-generation sequencing technology and clinical data of lung adenocarcinoma patients from the Cancer Genome Atlas showed that miR-181a-5p is one of the deregulated miRNAs in lung adenocarcinoma [28]. Regarding the expression of miR-630, miRNA expression profiling using microarray hybridization technology demonstrated that this miRNA is a possible candidate for the identification of NSCLC recurrence prediction [29]. Taken all together, the expression of miR-181a-5p and miR-630 is important and takes a part in lung cancer pathogenesis, therefore these miRNAs have a high potential of being used as lung cancer biomarkers, however, previous studies did not answer if these miRNAs could be used as non-invasive/low invasive biomarkers for lung cancer.

The aim of the present study was to investigate the expression level of miR-181a-5p and miR-630 in NSCLC tumor tissue and plasma samples; to investigate the association between miR-181a-5p, miR-630 expression and *BCL2, LMO3, PTEN, SNAI2, WIF1* expression and NSCLC progression and prognosis of patients; to analyze the association between these molecular factors and NSCLC patient response to treatment, disease progression and the survival of patients; and to evaluate the potential (including the potential of being used as non-invasive biomarkers) of molecular factors analyzed as the molecular markers of NSCLC.

## Methods

### Patients and clinical specimens

The study was performed in 89 paired tissue specimens (NSCLC tumors and paired noncancerous tissues) and plasma samples obtained from NSCLC patients before surgical treatment (anatomical segmentectomy, lobectomy, or pulmonectomy) at the Department of Thoracic Surgery and Oncology of the National Cancer Institute (Vilnius, Lithuania). The scope of surgery was chosen individually for each patient based on the location of the cancerous process and its spread. Plasma samples from 89 healthy individuals were also collected as normal controls. All patients were selected on the criteria that they hadn’t received pre-operative chemotherapy or radiotherapy. The present study was approved by the Lithuanian Bioethics Committee (No. 158200-05-455-141). Written informed consent was obtained from all patients prior to sample collection. The patient’s clinicopathological data was assessed from medical records at the same institution. After surgical treatment, all patients every 3 weeks received four courses of adjuvant combination therapy with platinum-based drug (cisplatin or carboplatin, respectively 80 mg/m2 intravenously and 5 AUC) and etoposide (120 mg/m2 intravenously) for further treatment. The adjuvant chemotherapy treatment was started 3–4 weeks after the end of the surgical treatment. The median follow-up period was 49 months (range, 1–98 months). Response to treatment had 49.4% (n = 44) patients and 50.6% (n = 45) were identified with disease progression. The clinicopathological characteristics of the study cohort are presented in Table 1.

**Table 1:** Clinicopathological characteristics of NSCLC patients

Characteristic	Cases, n (%)
Age	
≤ 68 > 68	47 (52.8) 42 (47.2)
Gender	
Female Male	12 (13.5) 77 (86.5)
Smoking status	
Never Smoking	35 (39.3) 54 (60.7)
Pathological stage	
Stage I/II Stage III/IV	65 (73.0) 24 (27.0)
Lymph node status	
N0 N1 N2	49 (55.1) 23 (25.8) 17 (19.1)
Tumor differentiation grade	
G1 G2 G3	5 (5.6) 30 (33.7) 54 (60.7)
Tumor histology	
ADC SCC	45 (50.6) 44 (49.4)
Response	
Stable disease Progression	44 (49.4) 45 (50.6)

N0 – no regional lymph nodes involvement; N1 – involvement of ipsilateral peribronchial and/or ipsilateral hilar lymph nodes (includes direct extension to intrapulmonary nodes); N2 – involvement of the ipsilateral mediastinal and/or subcarinal lymph nodes; G1 – well differentiated; G2 –moderately differentiated, G3 – poorly differentiated; ADC – adenocarcinoma; SCC – squamous cell carcinoma; HR – Hazard ratio, CI – confidence interval

### Gene expression analysis

All resected lung cancer tumors and paired noncancerous tissues were histologically reviewed by a pathologist, then immediately frozen in liquid nitrogen and stored at -150 °C. Plasma samples of NSCLC patients and control samples were frozen and stored at -70 °C. Total RNA from the tumor and adjacent normal tissues was extracted using mirVana™ miRNA isolation kit (Ambion, USA) according to the manufacturer’s instruction. miRNAs from NSCLC blood plasma and control samples were extracted using miRNeasy Serum/Plasma Kit (Qiagen, Germany).

Analysis of miR-181a-5p and miR-630 expression was performed by qRT-PCR using TaqMan miRNA specific primers (Applied Biosystems, USA). Whereas *BCL2, LMO3, PTEN, SNAI2, WIF1* expression levels were identified with KAPA SYBR FAST qPCR Kit (Kapa Biosystems, USA). For miRNAs expression analysis samples were normalized relative to the expression of housekeeping gene RNU6B, while for gene expression – to *β-ACTIN*. Each sample was examined in triplicate and calculated following the 2^−ΔΔCt^ method [30].

### Statistical analysis

The statistical analysis was either performed using the data analysis software package SPSS 20.0 (IBM Corp.) or GraphPad v8.0 (GraphPad Software, Inc.). Normal distribution of the data was verified using Shapiro–Wilk W test. Differences in the expression level between tumor and adjacent normal tissue were analyzed using a paired t-test, while differences in expression level between NSCLC plasma samples and healthy controls were analyzed using an unpaired t-test. Differential expression was examined by nonparametric Wilcoxon (for paired samples) and Mann–Whitney (for unpaired samples) tests. Patients based on the gene median expression (cut-off value) were separated into two groups. Samples expressing miR-181a-5p and miR-630 less than median expression were assigned to the low expression group; samples above the median value were assigned to the high expression group. A two-sided Chi-square test or Fisher’s exact test were used to analyze the distribution of cases with low or high miRNAs level in tumor and plasma samples according to the demographic and clinicopathological characteristics of the patients. The overall survival (OS) and progression-free survival (PFS) were evaluated by Kaplan-Meier analysis and log-rank test. Univariate analysis and multivariate Cox regression analysis were performed to detect independent factors significantly determining PFS or OS. When the p-value was less than 0.05, the differences were considered statistically significant.

## Results

### Expression level of miR-181a-5p and miR-630 in NSCLC

**Fig. 1:**
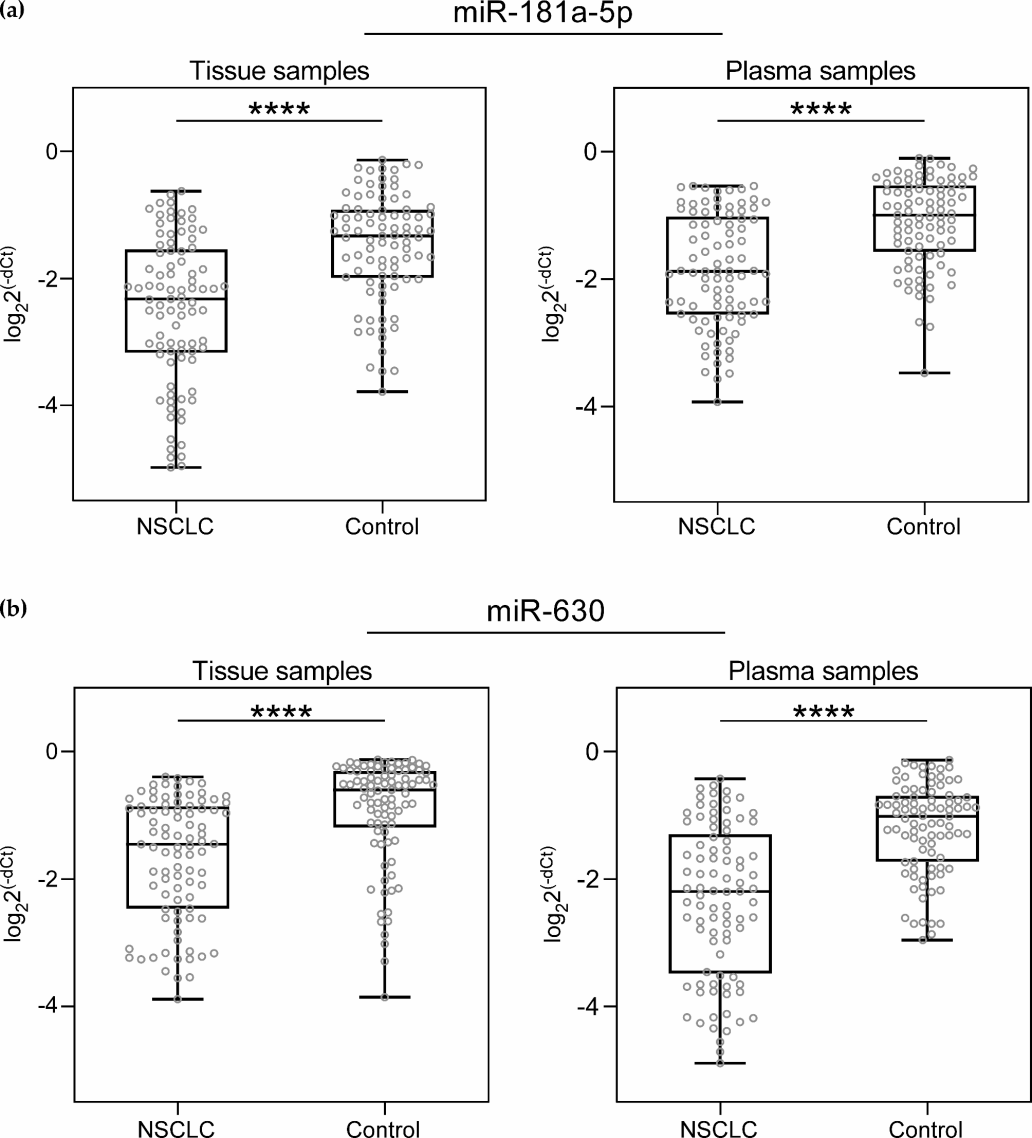
Relative miR-181a-5p (**A**) and miR-630 (**B**) expression in NSCLC tissue (n = 89) and plasma (n = 89) samples compared with healthy or adjacent normal controls detected by qRT-PCR. Expression levels of miRNAs were determined by the 2^−ΔCt^ method. Wilcoxon and Mann–Whitney tests were used to evaluate statistically significant differences. Whiskers of boxplot denote nonoutlier range. ****above box plots indicate the significant difference p < 0.0001

The expression levels of miR-181a-5p and miR-630 in 89 paired NSCLC tissue specimens and plasma samples were quantified by qRT-PCR. It was found that mRNA expression levels of miR-181a-5p in NSCLC tissue and plasma samples were significantly decreased compared with control samples, respectively (both p < 0.0001) (Fig. 1A). Similarly, the expression of miR-630 was also significantly downregulated in NSCLC tissue and plasma samples compared with healthy or adjacent normal controls, respectively (both p < 0.0001) (Fig. 1B).

### Distribution of cases with low or high miR-181a-5p, miR-630 level in tumor tissue according to demographic and clinicopathological characteristics of NSCLC patients

89 NSCLC tumors and paired noncancerous tissues were investigated. Changes in miR-181a-5p expression in tumor tissue were significantly associated with patients’ gender (p = 0.018), tumor differentiation grade (p = 0.048), and response to treatment (p = 0.013), while miR-630 expression despite the significant difference between tumor and adjacent healthy tissue did not show any statistically significant relationship to patients clinicopathological characteristics. Our results suggest that slower disease progression is expected for NSCLC patients with low miR-181a-5p expression in tumor tissue after surgery and cisplatin/etoposide treatment. The distribution between miR-181a-5p, miR-630 expression in tumor tissue and demographic, clinicopathological characteristics of the patients are presented in additional files (Additional file 1).

### Distribution of cases with low or high miR-181a-5p, miR-630 level in plasma according to demographic and clinicopathological characteristics of NSCLC patients

89 NSCLC patients’ plasma and healthy individuals control samples were investigated. Changes in miR-181a-5p expression in plasma samples were significantly associated with patients’ response to treatment (p = 0.022), while miR-630 expression was associated only with patients’ age (p = 0.007). Our results indicate that slower disease progression is expected for NSCLC patients with low miR-181a-5p expression in plasma after surgery and cisplatin/etoposide treatment. Moreover, miR-181a-5p may be used as a non-invasive predictive biomarker for NSCLC patients. The distribution between miR-181a-5p, miR-630 expression in plasma samples and demographic, clinicopathological characteristics of the patients are presented in additional files (Additional file 2).

### Association between miR-181a-5p, miR-630 expression, and NSCLC patient survival rates

**Fig. 2:**
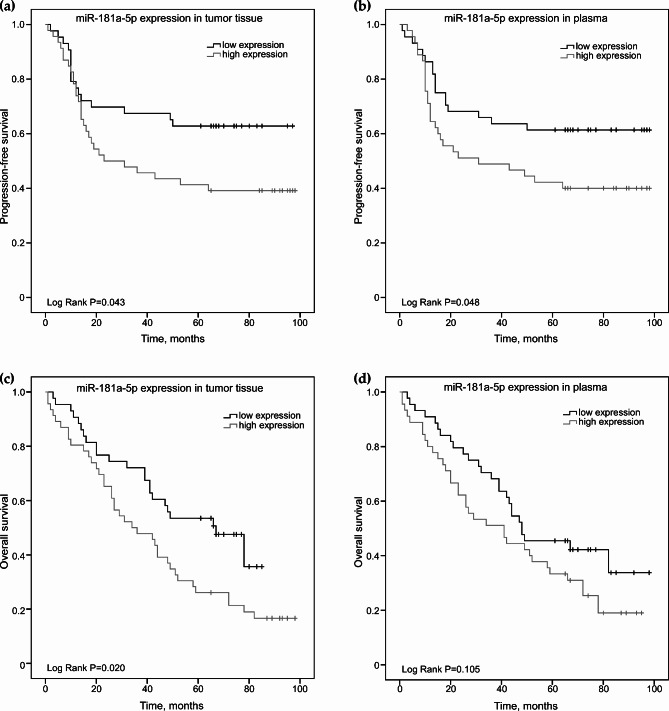
Kaplan Meier curves of progression-free (**A, B**) and overall survival (**C, D**) according to miR-181a-5p expression in NSCLC tumor tissue and plasma samples (n = 89). Patients were classified into high and low expression groups according to the miR-181a-5p median value. Curves were compared using the log-rank test, p values are shown

Next, the prognostic value of miR-181a-5p and miR-630 expression in NSCLC tumor tissues and plasma samples was investigated. The median time of OS and PFS was established and compared to each group using Kaplan-Meier analysis. Mean PFS survival for all NSCLC patients was 58.1 months (95% CI: 49.5–66.8), while mean survival rates without progression for NSCLC patients with low miR-181a-5p expression in tumor were 66.9 months (95% CI: 55.0-78.9) as well as in plasma ‒ 66.4 months (95% CI: 54.4–78.4). The analysis of the obtained data demonstrates that patients with low miR-181a-5p expression in tumor tissue (Fig. 2A, p = 0.043) and plasma (Fig. 2B, p = 0.048) had longer PFS rates than those with high miRNA expression. Moreover, mean OS survival for all group was 51.4 months (95% CI: 44.2–58.6), meanwhile mean OS rates for NSCLC patients with low miR-181a-5p expression in tumor were 55.8 months (95% CI: 46.9–64.8). Analysis showed, that decreased miR-181a-5p expression in tumor was significantly associated with longer OS rates (Fig. 2C, p = 0.020). No statistically significant relationship was observed between miR-181a-5p expression in plasma and NSCLC patients’ overall survival (Fig. 2D).

**Fig. 3:**
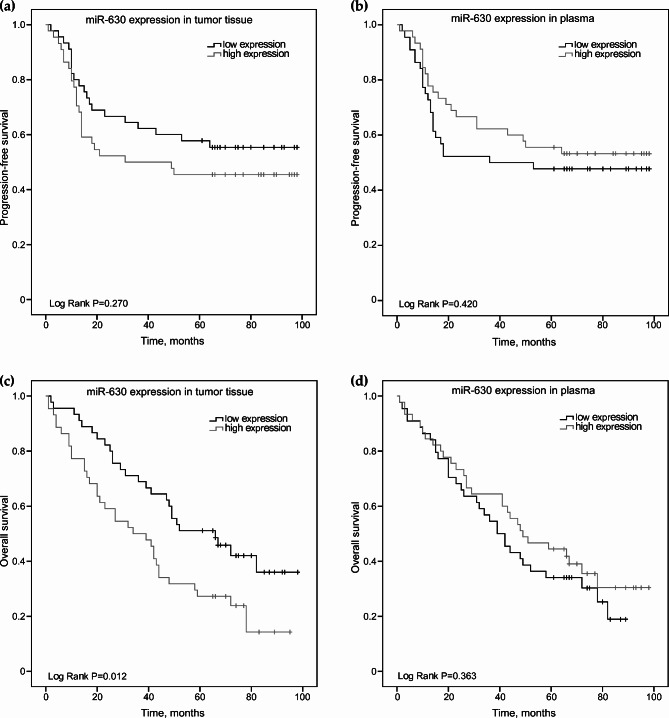
Kaplan Meier curves of progression-free (**A, B**) and overall survival (**C, D**) according to miR-630 expression in NSCLC tumor tissue and plasma samples (n = 89). Patients were classified into high and low expression groups according to the miR-630 median value. Curves were compared using the log-rank test, p values are shown

Also, mean OS survival for all group was 51.4 months (95% CI: 44.2–58.6), while mean OS rates for NSCLC patients with low miR-630 expression in tumor were 60.9 months (95% CI: 51.0-70.9). Expression of miR-630 in tumor (Fig. 3A) or plasma (Fig. 3B) samples did not show any significant relationship with NSCLC patients’ progression-free survival rates. Meanwhile, decreased miR-630 expression in tumor (Fig. 3C, p = 0.012) was statistically significantly associated with better NSCLC patients’ overall survival, whereas in plasma (Fig. 3D) significant relationship was not observed.

The prognostic factors for OS and PFS were subsequently analyzed using univariate and multivariate Cox regression analysis in NSCLC tissue (Tables 2 and 3) and plasma samples (Tables 4 and 5). Our results suggest that the expression of miR-181a-5p (HR: 2.1, 95% CI: 1.2–3.7, p = 0.009) as well as miR-630 (HR: 2.1, 95% CI: 1.2–3.6, p = 0.010) in tumor tissue are the statistically significant variables for NSCLC patients’ overall survival (Table 2). Moreover, in NSCLC patient plasma samples circulating miR-181a-5p can be evaluated as significant independent prognostic factors for overall survival and progression-free survival (respectively HR: 1.8, 95% CI: 1.0–3.0, p = 0.033; HR: 2.0, 95% CI: 1.1–3.9, p = 0.033) (Tables 4 and 5).

**Table 2:** Univariate and Multivariate Cox regression of prognostic factors for NSCLC patients’ OS in tumor tissue

Variables	Univariate		Multivariate for miR-181a-5p		Multivariate for miR-630	
	HR (95% CI)	*P*-value	HR (95% CI)	*P*-value	HR (95% CI)	*P*-value
Age						
≤ 68	Ref.		Ref.		Ref.	
> 68	1.0 (0.6–1.6)	0.853	1.0 (0.5–1.9)	0.952	1.1 (0.6-2.0)	0.855
Gender						
Male	Ref.		Ref.		Ref.	
Female	0.9 (0.5–1.9)	0.865	0.6 (0.3–1.4)	0.245	0.9 (0.4–2.2)	0.825
Smoking status						
Never	Ref.		Ref.		Ref.	
Smoking	1.0 (0.6–1.6)	0.917	1.3 (0.7–2.5)	0.465	1.4 (0.7–2.6)	0.374
Pathological stage						
Stage I/II	Ref.		Ref.		Ref.	
Stage III/IV	1.7 (1.0-2.9)	0.058	0.9 (0.3–2.7)	0.815	0.7 (0.2–2.2)	0.514
Lymph node status						
N0	Ref.		Ref.		Ref.	
N1	1.1 (0.6-2.0)	0.800	1.0 (0.5–2.1)	0.962	1.2 (0.6–2.4)	0.639
N2	**2.4 (1.3–4.6)**	**0.006**	3.4 (0.9–12.5)	0.068	**4.2 (1.1–15.9)**	**0.035**
Tumor differentiation grade						
G1	Ref.		Ref.		Ref.	
G2	0.8 (0.3–2.5)	0.755	0.6 (0.2–1.8)	0.358	0.6 (0.2–1.7)	0.304
G3	0.9 (0.3–2.5)	0.804	0.6 (0.2–1.9)	0.395	0.6 (0.2–1.9)	0.376
Histology						
ADC	Ref.		Ref.		Ref.	
SCC	1.2 (0.7-2.0)	0.473	1.2 (0.7–2.3)	0.539	1.1 (0.6–1.9)	0.877
miR-181a-5p in tumor						
low expression	Ref.		Ref.		—	—
high expression	**1.8 (1.1–3.1)**	**0.022**	**2.1 (1.2–3.7)**	**0.009**		
miR-630 in tumor						
low expression	Ref.		—	—	Ref.	
high expression	**1.9 (1.1–3.2)**	**0.014**			**2.1 (1.2–3.6)**	**0.010**

N0 – no regional lymph nodes involvement; N1 – involvement of ipsilateral peribronchial and/or ipsilateral hilar lymph nodes (includes direct extension to intrapulmonary nodes); N2 – involvement of the ipsilateral mediastinal and/or subcarinal lymph nodes; G1 – well differentiated; G2 –moderately differentiated, G3 – poorly differentiated; ADC – adenocarcinoma; SCC – squamous cell carcinoma; HR – Hazard ratio, CI – confidence interval; Ref. – reference group.

**Table 3:** Univariate and Multivariate Cox regression of prognostic factors for NSCLC patients’ PFS in tumor tissue

Variables	Univariate		Multivariate for miR-181a-5p		Multivariate for miR-630	
	HR (95% CI)	*P*-value	HR (95% CI)	*P*-value	HR (95% CI)	*P*-value
Age						
≤ 68	Ref.		Ref.		Ref.	
> 68	0.6 (0.4–1.2)	0.142	0.7 (0.3–1.5)	0.355	0.7 (0.3–1.5)	0.350
Gender						
Male	Ref.		Ref.		Ref.	
Female	**2.0 (1.0-4.1)**	**0.047**	1.0 (0.4–2.6)	0.979	1.3 (0.5–3.3)	0.568
Smoking status						
Never	Ref.		Ref.		Ref.	
Smoking	0.8 (0.5–1.5)	0.531	0.9 (0.4–1.9)	0.862	1.0 (0.5-2.0)	0.916
Pathological stage						
Stage I/II	Ref.		Ref.		Ref.	
Stage III/IV	**2.5 (1.4–4.6)**	**0.003**	1.7 (0.6–4.6)	0.316	1.6 (0.6–4.7)	0.362
Lymph node status						
N0	Ref.		Ref.		Ref.	
N1	0.7 (0.3–1.5)	0.331	0.6 (0.3–1.4)	0.246	0.7 (0.3–1.6)	0.340
N2	**2.8 (1.4–5.5)**	**0.003**	1.7 (0.5–5.9)	0.276	1.7 (0.5-6.0)	0.382
Tumor differentiation grade						
G1	Ref.		Ref.		Ref.	
G2	2.3 (0.5–10.0)	0.262	1.7 (0.4–7.9)	0.501	1.8 (0.4–8.3)	0.464
G3	1.6 (0.4–6.6)	0.545	1.2 (0.2–5.6)	0.857	1.2 (0.3–5.8)	0.815
Histology						
ADC	Ref.		Ref.		Ref.	
SCC	0.7 (0.4–1.3)	0.263	0.9 (0.4–1.7)	0.663	0.8 (0.4–1.6)	0.554
miR-181a-5p in tumor						
low expression	Ref.		Ref.		—	—
high expression	**1.9 (1.0-3.5)**	**0.046**	1.8 (0.9–3.5)	0.083		
miR-630 in tumor						
low expression	Ref.		—	—	Ref.	
high expression	1.3 (0.7–2.4)	0.364			1.4 (0.7–2.6)	0.349

N0 – no regional lymph nodes involvement; N1 – involvement of ipsilateral peribronchial and/or ipsilateral hilar lymph nodes (includes direct extension to intrapulmonary nodes); N2 – involvement of the ipsilateral mediastinal and/or subcarinal lymph nodes; G1 – well differentiated; G2 –moderately differentiated, G3 – poorly differentiated; ADC – adenocarcinoma; SCC – squamous cell carcinoma; HR – Hazard ratio, CI – confidence interval; Ref. – reference group.

**Table 4:** Univariate and Multivariate Cox regression of prognostic factors for NSCLC patients’ OS in plasma

Variables	Univariate		Multivariate for miR-181a-5p		Multivariate for miR-630	
	HR (95% CI)	*P*-value	HR (95% CI)	*P*-value	HR (95% CI)	*P*-value
Age						
≤ 68	Ref.		Ref.		Ref.	
> 68	1.0 (0.6–1.6)	0.853	1.1 (0.6–1.9)	0.855	0.9 (0.5–1.8)	0.864
Gender						
Male	Ref.		Ref.		Ref.	
Female	0.9 (0.5–1.9)	0.865	0.6 (0.3–1.4)	0.270	0.8 (0.3–1.8)	0.566
Smoking status						
Never	Ref.		Ref.		Ref.	
Smoking	1.0 (0.6–1.6)	0.917	1.4 (0.7–2.6)	0.352	1.3 (0.6–2.5)	0.496
Pathological stage						
Stage I/II	Ref.		Ref.		Ref.	
Stage III/IV	1.7 (1.0-2.9)	0.058	1.0 (0.4-3.0)	0.965	1.0 (0.3–2.8)	0.950
Lymph node status						
N0	Ref.		Ref.		Ref.	
N1	1.1 (0.6-2.0)	0.800	1.0 (0.5–1.9)	0.922	1.0 (0.5–2.1)	0.913
N2	**2.4 (1.3–4.6)**	**0.006**	3.2 (0.9–10.9)	0.069	3.0 (0.8–10.5)	0.090
Tumor differentiation grade						
G1	Ref.		Ref.		Ref.	
G2	0.8 (0.3–2.5)	0.755	0.7 (0.2–2.1)	0.508	0.7 (0.2–2.1)	0.506
G3	0.9 (0.3–2.5)	0.804	0.5 (0.2–1.8)	0.312	0.6 (0.2–1.8)	0.339
Histology						0.600
ADC	Ref.		Ref.		Ref.	
SCC	1.2 (0.7-2.0)	0.473	1.2 (0.6–2.1)	0.605	1.2 (0.6–2.1)	
miR-181a-5p in plasma						
low expression	Ref.		Ref.		—	—
high expression	1.5 (0.9–2.5)	0.110	**1.8 (1.0–3.0)**	**0.033**		
miR-630 in plasma						
low expression	Ref.		—	—	Ref.	
high expression	0.8 (0.5–1.3)	0.368			0.8 (0.4–1.3)	0.337

N0 – no regional lymph nodes involvement; N1 – involvement of ipsilateral peribronchial and/or ipsilateral hilar lymph nodes (includes direct extension to intrapulmonary nodes); N2 – involvement of the ipsilateral mediastinal and/or subcarinal lymph nodes; G1 – well differentiated; G2 –moderately differentiated, G3 – poorly differentiated; ADC – adenocarcinoma; SCC – squamous cell carcinoma; HR – Hazard ratio, CI – confidence interval; Ref. – reference group.

**Table 5:** Univariate and Multivariate Cox regression of prognostic factors for NSCLC patients’ PFS in plasma

Variables	Univariate		Multivariate for miR-181a-5p		Multivariate for miR-630	
	HR (95% CI)	*P*-value	HR (95% CI)	*P*-value	HR (95% CI)	*P*-value
Age						
≤ 68	Ref.		Ref.		Ref.	
> 68	0.6 (0.4–1.2)	0.142	0.8 (0.4–1.7)	0.609	0.6 (0.3–1.3)	0.168
Gender						
Male	Ref.		Ref.		Ref.	
Female	**2.0 (1.0-4.1)**	**0.047**	1.0 (0.4–2.5)	0.918	1.4 (0.6–3.4)	0.481
Smoking status						
Never	Ref.		Ref.		Ref.	
Smoking	0.8 (0.5–1.5)	0.531	1.0 (0.5-2.0)	0.940	0.9 (0.4-2.0)	0.827
Pathological stage						
Stage I/II	Ref.		Ref.		Ref.	
Stage III/IV	**2.5 (1.4–4.6)**	**0.003**	2.0 (0.7–5.4)	0.198	1.6 (0.6–4.5)	0.343
Lymph node status						
N0	Ref.		Ref.		Ref.	
N1	0.7 (0.3–1.5)	0.331	0.6 (0.3–1.4)	0.255	0.6 (0.3–1.5)	0.294
N2	**2.8 (1.4–5.5)**	**0.003**	1.7 (0.5–5.4)	0.386	1.7 (0.5–5.5)	0.393
Tumor differentiation grade						
G1	Ref.		Ref.		Ref.	
G2	2.3 (0.5–10.0)	0.262	2.1 (0.4–9.7)	0.353	2.1 (0.4–9.7)	0.350
G3	1.6 (0.4–6.6)	0.545	1.2 (0.2–5.7)	0.852	1.3 (0.3–6.6)	0.724
Histology						
ADC	Ref.		Ref.		Ref.	
SCC	0.7 (0.4–1.3)	0.263	0.8 (0.4–1.6)	0.510	0.8 (0.4–1.5)	0.416
miR-181a-5p in plasma						
low expression	Ref.		Ref.		—	—
high expression	**1.9 (1.0-3.4)**	**0.042**	**2.0 (1.1–3.9)**	**0.033**		
miR-630 in plasma						
low expression	Ref.		—	—	Ref.	
high expression	0.8 (0.4–1.4)	0.359			0.6 (0.3–1.1)	0.086

N0 – no regional lymph nodes involvement; N1 – involvement of ipsilateral peribronchial and/or ipsilateral hilar lymph nodes (includes direct extension to intrapulmonary nodes); N2 – involvement of the ipsilateral mediastinal and/or subcarinal lymph nodes; G1 – well differentiated; G2 –moderately differentiated, G3 – poorly differentiated; ADC – adenocarcinoma; SCC – squamous cell carcinoma; HR – Hazard ratio, CI – confidence interval; Ref. – reference group.

### Relationship between *BCL2*, *LMO3*, *PTEN*, *SNAI2*, *WIF1* expression and clinicopathological characteristics of NSCLC patients in tumor tissue

To find an association between miR-181a-5p and miR-630 target genes *(BCL2*, *LMO3*, *PTEN*, *SNAI2*, *WIF1*), 89 cases of NSCLC and matching adjacent normal samples were investigated. All patients were divided into those with low or high gene expression based on the median *BCL2*, *LMO3*, *PTEN*, *SNAI2*, *WIF1* expression level. Statistically significant differences were found only between *SNAI2* expression and NSCLC patients’ tumor histology (p = 0.035) and lymph nodes status (p = 0.048). The distribution between *BCL2*, *LMO3*, *PTEN*, *SNAI2*, *WIF1* expression in tumor tissue and demographic, clinicopathological characteristics of the patients are presented in additional files (Additional file 3). Moreover, Cox regression analysis of progression-free and overall survival according to *BCL2*, *LMO3*, *PTEN*, *SNAI2*, *WIF1* expression in NSCLC tumor tissue did not show any statistically significant associations (data not shown).

## Discussion

The identification of cancer-specific miRNAs is significant for the accumulation of new cancer biomarkers. Furthermore, functional characterization of these miRNAs is important for the understanding of cancer biology. Various studies have shown that changes in miRNA expression play an essential role in human malignancies [31]. Identification of cancer‑related miRNAs and their target genes is essential for understanding the process of tumorigenesis and may be important for the development of novel therapeutic targets [32].

The present study showed that low miR-181a-5p expression in tumor is associated with NSCLC patients’ gender, tumor differentiation grade, response to treatment as well as better progression-free and overall survival rates. While in NSCLC patients’ plasma samples, low miR-181a-5p expression is significant for the evaluation of response to treatment and can be evaluated as significantly independent prognostic factors for progression-free and overall survival. Specific gender variability in miRNA expression is described in several diseases, mostly in those with some gender-related predisposition. Regulation of miRNA expression may be affected by gender-dependent factors, like sex steroid hormones or X-linked genes [33]. In addition, Cui et al. [34] functional enrichment analysis of miRNAs expression showed that female miRNA genes are significantly related to metabolism and cell cycle processes, whereas male miRNA genes are associated with histone modification and circadian rhythm. Pirlog et al. [12] showed that miR-181a-5p in addition takes part in epithelial-to-mesenchymal transition (EMT) regulation. Study results demonstrate, that EMT is an essential process for tumor cells to receive invasive and metastatic potential [35], therefore miR-181a-5p, despite its gender-associated expression is very likely to have potential as a diagnostic and prognostic biomarker in lung cancer. Moreover, the expression of miR-181a-5p was significantly downregulated in tumor tissue of three main histological subtypes (lung squamous cell carcinoma, lung adenocarcinoma, neuroendocrine lung cancer), suggesting that miR-181a-5p suppression is an important process in all types of lung cancer that is necessary for tumor initiation and progression.

Nevertheless, our results indicate the lower expression of miR-181a-5p and miR-630 in NSCLC tumors is associated with the longer PFS and OS of the patients. In addition, the lower expression of circulating miR-181a-5p (plasma samples) is as well associated with longer PFS and OS of surgery and cisplatin/etoposide treated NSCLC patients. Therefore, despite the downregulation of miR-181a-5p and miR-630 being associated with the development of cancer, the downregulation of miR-181a and miR-630 results in the sensitivity of cancer cells to the surgery/cisplatin/etoposide treatment or the slower disease progression. The disagreement to the previous observation that the high expression of miR-181a-5p increases the cisplatin sensitivity of A549 lung cancer cells by suppressing MAPK/Slug pathway and regulates EMT by targeting *PTEN* [36, 37] perhaps could be attributed to the specificity of the model system used.

As far as we know, there are no published reports concerning the relationship between miR-181a-5p expression and NSCLC treatment with cisplatin and etoposide prediction in tissue or plasma samples. On a cellular level, it is shown that miR-181a-5p is associated with gefitinib resistance in the PC9GR lung cancer cell line through direct targeting of *GAS7*, which is involved in the regulation of AKT/ERK pathways [38]. Overall, our study results indicate that NSCLC patients with low miR-181a-5p expression tend to have better progression-free and overall survival than patients with high tumor miR-181a-5p expression. Furthermore, low expression of circulating miR-181a-5p in surgery/cisplatin/etoposide treated NSCLC patient blood (plasma samples) is associated with more prolonged progression-free and overall survival of the patient, suggesting its potential as a non-invasive predictive and prognostic biomarker. Moreover, this result is important in the field of preoperative medicine as a predictive biomarker and could be included in the standardized protocols for data analysis as relevant molecular biomarker for the clinical routine in the near future.

However, results from Xue et al. [26] showed that NSCLC patients with high miR-181a-5p expression had a longer overall survival time than those with low miR-181a-5p expression in plasma. In support to our findings, Papadaki et al. [39] showed that patients with diagnosed squamous cell carcinoma and miR-181a-5p overexpression were associated with worse overall survival rates. Generally, different functions of miR‑181a-5p in carcinogenesis may be associated with its different target genes, depending on the tissue or cell microenvironment. Our results indicate that miR-181a-5p may play an essential role in the molecular pathogenesis and prognosis of NSCLC and therefore is likely to have potential as NSCLC biomarker.

The role of miR-630 in NSCLC is not well understood. In our study, we found that miR-630 expression in NSCLC tumor tissue can be used as a significantly independent prognostic factor for the patient’s overall survival. Any relationship between miR-630 expression and NSCLC treatment outcome has not been found. It is shown that miR-630 expression levels are related to gefitinib resistance via miR-630/YAP1/ERK feedback loop in EGFR-mutated lung adenocarcinoma cells [40]. Chen et al. [41] suggest that NSCLC patients with low miR-630 and high *BCL2* expression had the shortest overall survival and progression-free survival rates compared to other groups. Furthermore, low miR-630 expression may confer cisplatin resistance and promote colony formation by modulating the apoptotic pathway via targeting 3′-UTR of *BCL2* in NSCLC cells. Song et al. [42] demonstrated that miR-630 expression was significantly down-regulated in NCI-H23, A549, H157, and H1299 NSCLC cell lines as well as in NSCLC tissues compared with matched adjacent normal tissues indicating a critical role of miR-630 in the progression of NSCLC. High miR-630 expression is associated with inhibited cell proliferation, migration, and invasion processes in NSCLC cells due to down-regulating of its direct target *LMO3* expression, which acts as an essential regulator of cell growth as well as interacts with the tumor suppressor p53 and regulates its function [42]. In addition, miR-630 may be involved in A549 cell apoptosis as it targets the cell proliferation regulator serine-threonine CDC7 kinase, which is essential for the initiation of DNA replication, and the apoptotic modulator EP300 to promote p53 protein instability [43].

Moreover, in our study, we investigated the association between miR-181a-5p and miR-630 target genes (*BCL2*, *LMO3*, *PTEN*, *SNAI2*, *WIF1*) expression and clinicopathological characteristics as well as survival rates of NSCLC patients in tumor tissue. Statistically significant distribution was found only between *SNAI2* expression and NSCLC patients’ tumor histology and lymph nodes status. miRNA target analysis was performed using bioinformatic analysis and literature search. One of the reasons that a statistically reliable result was not obtained is the limited sample size. Our research aims to understand the complex relationships between the various factors and covariates and their impact on the time until a decisive event occurs. While recognizing the inherent limitations associated with a sample size of 89 individuals, we were aware of the risks of overfitting and instability in parameter estimates that can arise from introducing more complex models. Also, as the sample size increases, there is potential for greater distinction in the miRNA expression levels during the survival period. The many factors - nine in total - integrated into our multiple Cox model further complicated the analysis. However, its relatively simple parameter estimation and its ability to handle censored data are harmoniously compatible with the complexity of our research design. Additional studies with larger sample sizes must be performed to achieve a statistically significant result.

## Conclusions

Taken all together, the knowledge in the field of personalized medicine based on the unique molecular biomarkers of each tumor is advancing. Our findings show that miR-181a-5p and miR-630 expression levels have the potential to predict and improve the treatment outcome and prognosis of NSCLC patients. Moreover, circulating miR-181a-5p have potential therapeutic value as a non-invasive biomarker for NSCLC.

### Electronic supplementary material

Below is the link to the electronic supplementary material.

Supplementary Material 1

## Data Availability

The datasets used and/or analysed during the current study are available from the corresponding author on reasonable request.

## Abbreviations

NSCLC: Non-small cell lung cancer
OS: Overall survival
PFS: Progression-free survival
N0: No regional lymph nodes involvement
N1: Involvement of ipsilateral peribronchial and/or ipsilateral hilar lymph nodes (includes direct extension to intrapulmonary nodes)
N2: Involvement of the ipsilateral mediastinal and/or subcarinal lymph nodes
G1: Well differentiated tumor
G2: Moderately differentiated tumor
G3: Poorly differentiated tumor
ADC: Adenocarcinoma
SCC: Squamous cell carcinoma
HR: Hazard ratio
CI: Confidence interval
Ref.: Reference group
