# Supplementary material for: Potential of miR-181a-5p and miR-630 as clinical biomarkers in NSCLC
Source: BMC Cancer. 2023 Sep 12;23:857. doi: 10.1186/s12885-023-11365-5 (PMC10496384; doi:10.1186/s12885-023-11365-5)
Supplement: Supplementary file 1 — Supplementary Material 1 [file 12885_2023_11365_MOESM1_ESM.docx]

**Additional file 1.** Distribution of cases with low or high miR-181a-5p, miR-630 level in tumor tissue according to demographic and clinicopathological characteristics of the patients

| Characteristic | miR-181a-5p expression | | p | miR-630 expression | | p |
| --- | --- | --- | --- | --- | --- | --- |
|  | **Low, n (%)** | **High, n (%)** |  | **Low, n (%)** | **High, n (%)** |  |
| Age  ≤ 68 years  > 68 years | 24 (55.8)  19 (44.2) | 23 (50.0)  23 (50.0) | 0.368 | 26 (57.8)  19 (42.2) | 21 (47.7)  23 (52.3) | 0.231 |
| Gender  Female  Male | 2 (4.7)  41 (95.3) | 10 (21.7)  36 (78.3) | **0.018** | 8 (17.8)  37 (82.2) | 4 (9.1)  40 (90.9) | 0.187 |
| Smoking status  Never  Smoking | 15 (34.9)  28 (65.1) | 20 (43.5)  26 (56.5) | 0.270 | 17 (37.8)  28 (62.2) | 18 (40.9)  26 (59.1) | 0.466 |
| Pathological stage  Stage I/II  Stage III/IV | 31 (72.1)  12 (27.9) | 34 (73.9)  12 (26.1) | 0.518 | 35 (77.8)  10 (22.2) | 30 (68.2)  14 (31.8) | 0.218 |
| Histology  ADC  SCC | 20 (46.5)  23 (53.5) | 25 (54.3)  21 (45.7) | 0.299 | 27 (60.0)  18 (40.0) | 18 (40.9)  26 (59.1) | 0.056 |
| Tumor differentiation grade  G1  G2  G3 | 3 (7.0)  9 (20.9)  31 (72.1) | 2 (4.3)  21 (45.7)  23 (50.0) | **0.048** | 3 (6.7)  13 (28.9)  29 (64.4) | 2 (4.5)  17 (38.6)  25 (56.9) | 0.601 |
| Lymph node status  N0  N1  N2 | 24 (55.8)  10 (23.3)  9 (20.9) | 25 (54.3)  13 (28.3)  8 (17.4) | 0.831 | 25 (55.6)  11 (24.4)  9 (20.0) | 24 (54.5)  12 (27.3)  8 (18.2) | 0.946 |
| Response  Stable disease  Progression | 27 (62.8)  16 (37.2) | 17 (37.0)  29 (63.0) | **0.013** | 24 (53.3)  21 (46.7) | 20 (45.5)  24 (54.5) | 0.298 |

N0 – no regional lymph nodes involvement; N1 – involvement of ipsilateral peribronchial and/or ipsilateral hilar lymph nodes (includes direct extension to intrapulmonary nodes); N2 – involvement of the ipsilateral mediastinal and/or subcarinal lymph nodes; G1 – well differentiated; G2 –moderately differentiated, G3 – poorly differentiated; ADC – adenocarcinoma; SCC – squamous cell carcinoma; HR – Hazard ratio, CI – confidence interval; Ref. – reference group.

**Additional file 2.** Distribution of cases with low or high miR-181a-5p, miR-630 level in plasma according to demographic and clinicopathological characteristics of the patients

| Characteristic | miR-181a-5p expression | | p | miR-630 expression | | p |
| --- | --- | --- | --- | --- | --- | --- |
|  | **Low, n (%)** | **High, n (%)** |  | **Low, n (%)** | **High, n (%)** |  |
| Age  ≤ 68 years  > 68 years | 23 (52.3)  21 (47.7) | 24 (53.3)  21 (46.7) | 0.545 | 17 (38.6)  27 (61.4) | 30 (66.7)  15 (33.3) | **0.007** |
| Gender  Female  Male | 4 (9.1)  40 (90.9) | 8 (17.8)  37 (82.2) | 0.187 | 4 (9.1)  40 (90.9) | 8 (17.8)  37 (82.2) | 0.187 |
| Smoking status  Never  Smoking | 15 (34.1)  29 (65.9) | 20 (44.4)  25 (55.6) | 0.217 | 20 (45.5)  24 (54.5) | 15 (33.3)  30 (66.7) | 0.170 |
| Pathological stage  Stage I/II  Stage III/IV | 30 (68.2)  14 (31.8) | 35 (77.8)  10 (22.2) | 0.218 | 30 (68.2)  14 (31.8) | 35 (77.8)  10 (22.2) | 0.218 |
| Histology  ADC  SCC | 23 (52.3)  21 (47.7) | 22 (48.9)  23 (51.1) | 0.457 | 20 (45.5)  24 (54.5) | 25 (55.6)  20 (44.4) | 0.229 |
| Tumor differentiation grade  G1  G2  G3 | 3 (6.8)  14 (31.8)  27 (61.4) | 2 (4.4)  16 (35.6)  27 (60.0) | 0.851 | 3 (6.8)  15 (34.1)  26 (59.1) | 2 (4.4)  15 (33.3)  28 (62.3) | 0.877 |
| Lymph node status  N0  N1  N2 | 23 (52.3)  11 (25.0)  10 (22.7) | 26 (57.8)  12 (26.7)  7 (15.5) | 0.689 | 23 (52.3)  12 (27.3)  9 (20.5) | 26 (57.8)  11 (24.4)  8 (17.8) | 0.872 |
| Response  Stable disease  Progression | 27 (61.4)  17 (38.6) | 17 (37.8)  28 (62.2) | **0.022** | 20 (45.5)  24 (54.5) | 24 (53.3)  21 (46.7) | 0.298 |

N0 – no regional lymph nodes involvement; N1 – involvement of ipsilateral peribronchial and/or ipsilateral hilar lymph nodes (includes direct extension to intrapulmonary nodes); N2 – involvement of the ipsilateral mediastinal and/or subcarinal lymph nodes; G1 – well differentiated; G2 –moderately differentiated, G3 – poorly differentiated; ADC – adenocarcinoma; SCC – squamous cell carcinoma; HR – Hazard ratio, CI – confidence interval; Ref. – reference group.

**Additional file 3.** Distribution of cases with low or high *BCL2*, *LMO3*, *PTEN*, *SNAI2*, *WIF1* level in tumor tissue according to demographic and clinicopathological characteristics of the patients

| Characteristic | *BCL2* | | p | *LMO3* | | p | *PTEN* | | p | *SNAI2* | | p | *WIF1* | | p |
| --- | --- | --- | --- | --- | --- | --- | --- | --- | --- | --- | --- | --- | --- | --- | --- |
|  | **Low, n (%)** | **High, n (%)** |  | **Low, n (%)** | **High, n (%)** |  | **Low, n (%)** | **High, n (%)** |  | **Low, n (%)** | **High, n (%)** |  | **Low, n (%)** | **High, n (%)** |  |
| Age  ≤ 68 years  > 68 years | 25 (55.6)  20 (44.4) | 22 (50.0)  22 (50.0) | 0.377 | 25 (56.8)  19 (43.2) | 22 (48.9)  23 (51.1) | 0.296 | 23 (51.1)  22 (48.9) | 24 (54.5)  20 (45.5) | 0.455 | 25 (56.8)  19 (43.2) | 22 (48.9)  23 (51.1) | 0.296 | 25 (55.6)  20 (44.4) | 22 (50.0)  22 (50.0) | 0.377 |
| Gender  Female  Male | 4 (8.9)  41 (91.1) | 8 (18.2)  36 (81.8) | 0.166 | 5 (11.4)  39 (88.6) | 7 (15.6)  38 (84.4) | 0.395 | 4 (8.9)  41 (91.1) | 8 (18.2)  36 (81.8) | 0.166 | 8 (18.2)  36 (81.8) | 4 (8.9)  41 (91.1) | 0.166 | 7 (15.6)  38 (84.4) | 5 (11.4)  39 (88.6) | 0.395 |
| Smoking status  Never  Smoking | 16 (35.6)  29 (64.4) | 19 (43.2)  25 (56.8) | 0.302 | 18 (40.9)  26 (59.1) | 17 (37.8)  28 (62.2) | 0.466 | 14 (31.1)  31 (68.9) | 21 (47.7)  23 (52.3) | 0.082 | 20 (45.5)  24 (54.5) | 15 (33.3)  30 (66.7) | 0.170 | 21 (46.7)  24 (53.3) | 14 (31.8)  30 (68.2) | 0.112 |
| Pathological stage  Stage I/II  Stage III/IV | 32 (71.1)  13 (28.9) | 33 (75.0)  11 (25.0) | 0.431 | 31 (70.5)  13 (29.5) | 34 (75.6)  11 (24.4) | 0.381 | 34 (75.6)  11 (24.4) | 31 (70.5)  13 (29.5) | 0.381 | 34 (77.3)  10 (22.7) | 31 (68.9)  14 (31.1) | 0.257 | 31 (68.9)  14 (31.1) | 34 (77.3)  10 (22.7) | 0.257 |
| Lymph node status  N0  N1  N2 | 25 (55.6)  10 (22.2)  10 (22.2) | 24 (54.5)  13 (29.5)  7 (16.0) | 0.628 | 23 (52.3)  13 (29.5)  8 (18.2) | 26 (57.8)  10 (22.2)  9 (20.0) | 0.732 | 25 (55.6)  12 (26.7)  8 (17.7) | 24 (54.5)  11 (25.0)  9 (20.5) | 0.946 | 30 (68.2)  8 (18.2)  6 (13.6) | 19 (42.2)  15 (33.3)  11 (24.5) | **0.048** | 24 (53.3)  11 (24.4)  10 (22.3) | 25 (56.8)  12 (27.3)  7 (15.9) | 0.747 |
| Differentiation grade  G1  G2  G3 | 4 (8.8)  16 (35.6)  25 (55.6) | 1 (2.3)  14 (31.8)  29 (65.9) | 0.330 | 3 (6.8)  16 (36.4)  25 (56.8) | 2 (4.4)  14 (31.1)  29 (64.4) | 0.734 | 2 (4.4)  19 (42.2)  24 (53.3) | 3 (6.8)  11 (25.0)  30 (68.2) | 0.224 | 2 (4.5)  12 (27.3)  30 (68.2) | 3 (6.7)  18 (40.0)  24 (53.3) | 0.358 | 5 (11.1)  13 (28.9)  27 (60.0) | 0 (0)  17 (38.6)  27 (61.4) | 0.063 |
| Histology  ADC  SCC | 24 (53.3)  21 (46.7) | 21 (47.7)  23 (52.3) | 0.376 | 22 (50.0)  22 (50.0) | 23 (51.1)  22 (48.9) | 0.543 | 25 (55.6)  20 (44.4) | 20 (45.5)  24 (54.5) | 0.229 | 27 (61.4)  17 (38.6) | 18 (40.0)  27 (60.0) | **0.035** | 24 (53.3)  21 (46.7) | 21 (47.7)  23 (52.3) | 0.376 |
| Response  Stable disease  Progression | 24 (53.3)  21 (46.7) | 20 (45.5)  24 (54.5) | 0.298 | 22 (50.0)  22 (50.0) | 22 (48.9)  23 (51.1) | 0.543 | 25 (55.6)  20 (44.4) | 19 (43.2)  25 (56.8) | 0.170 | 21 (47.7)  23 (52.3) | 23 (51.1)  22 (48.9) | 0.457 | 21 (46.7)  24 (53.3) | 23 (52.3)  21 (47.7) | 0.376 |

N0 – no regional lymph nodes involvement; N1 – involvement of ipsilateral peribronchial and/or ipsilateral hilar lymph nodes (includes direct extension to intrapulmonary nodes); N2 – involvement of the ipsilateral mediastinal and/or subcarinal lymph nodes; G1 – well differentiated; G2 –moderately differentiated, G3 – poorly differentiated; ADC – adenocarcinoma; SCC – squamous cell carcinoma; HR – Hazard ratio, CI – confidence interval; Ref. – reference group.
